# Main Complications during Pregnancy and Recommendations for Adequate Antenatal Care in Sickle Cell Disease: A Literature Review

**DOI:** 10.1055/s-0042-1742314

**Published:** 2022-02-09

**Authors:** Camilla Olivares Figueira, Fernanda Garanhani Surita, Kleber Fertrin, Guilherme de Moraes Nobrega, Maria Laura Costa

**Affiliations:** 1 Department of Obstetrics and Gynecology, Universidade Estadual de Campinas, Campinas, SP, Brazil; 2 Division of Hematology, Department of Medicine, University of Washington, Seattle, Washington, United States

**Keywords:** sickle cell disease, maternal morbidity, maternal mortality, pregnancy, anemia falciforme, morbidade materna, mortalidade materna, gestação

## Abstract

Sickle cell disease (SCD) is the most common monogenic disease worldwide, with a variable prevalence in each continent. A single nucleotide substitution leads to an amino-acid change in the β-globin chain, altering the normal structure ofhemoglobin, which is then called hemoglobin S inherited in homozygosity (HbSS) or double heterozygosity (HbSC, HbSβ), and leads to chronic hemolysis, vaso-occlusion, inflammation, and endothelium activation. Pregnant women with SCD are at a higher risk of developing maternal and perinatal complications. We performed a narrative review of the literature considering SCD and pregnancy, the main clinical and obstetrical complications, the specific antenatal care, and the follow-up for maternal and fetal surveillance. Pregnant women with SCD are at a higher risk of developing clinical and obstetric complications such as pain episodes, pulmonary complications, infections, thromboembolic events, preeclampsia, and maternal death. Their newborns are also at an increased risk of developing neonatal complications: fetal growth restriction, preterm birth, stillbirth. Severe complications can occur in patients of any genotype. We concluded that SCD is a high-risk condition that increases maternal and perinatal morbidity and mortality. A multidisciplinary approach during pregnancy and the postpartum period is key to adequately diagnose and treat complications.

## Resumo

Doença falciforme (DF) é a condição genética mais comum no mundo, com uma prevalência variável nos continentes. A substituição de um nucleotídeo muda um aminoácido na cadeia da β globina, e altera a estrutura normal da hemoglobina, que é então chamada de hemoglobina S, e pode ser herdada em homozigose (HbSS) ou heterozigose (HbSC, HbSβ), e leva a hemólise crônica, vaso-oclusão, inflamação, e ativação endotelial. Realizou-se uma revisão narrativa da literatura considerando doença falciforme e gestação, as complicações clínicas e obstétricas, o cuidado antenatal específico, e o seguimento para monitoramento materno e fetal. Gestantes com DF têm maior risco de desenvolver complicações clínicas e obstétricas, como crises dolorosas, complicações pulmonares, infecções, eventos tromboembólicos, pré-eclâmpsia, e morte materna. E seus recém-nascidos correm maior risco de desenvovler complicações neonatais: restrição de crescimento fetal, prematuridade e óbito fetal/neonatal. Complicações graves podem ocorrer em qualquer genótipo da doença. Concluiu-se que DF é uma condição de alto risco que aumenta a morbimortalidade materna e perinatal. Um seguimento com abordagem multidisciplinar na gestação e puerpério é fundamental para o diagnóstico e o tratamento das complicações.

## Introduction

Sickle cell disease (SCD) is the most common monogenic recessive inherited disease worldwide, and it was first described more than a century ago. 1 Approximately 300 thousand children are born with the disease every year, 2 3 4 and the prevalence of the mutated gene varies in each continent. In the Americas, the prevalence is of about 0.49 for every one thousand live births, 0.07 in Europe, 0.68 in South and Southeast Asia, and 10.68 in Africa. 5 In Brazil, there are 2 million people carrying the sickle-cell gene mutation, and about 25 thousand to 50 thousand people with the homozygous form of the disease. 6 The incidence in Brazil varies according to the state, from 1:650 live births in Bahia to 1:13,500 live births in Rio Grande do Sul. 7

A single nucleotide substitution leading to a switch in a glutamic acid residue to a valine one in the β globin chain turns normal hemoglobin into a structurally-abnormal one, called hemoglobin S (HbS), which causes SCD and is associated to endothelium activation and chronic inflammation. Other genotypes include hemoglobinopathy SC (HbSC), when the mutated hemoglobin S is associated with hemoglobin C, and sickle beta thalassemia, when hemoglobin S is associated with thalassemia mutations in the β chain (HbSβ). 1 2 8 9 Those heterozygous forms may have better outcomes, 10 11 although they are also associated with higher morbidity and mortality, especially during pregnancy and the postpartum period.

Complications in pregnancy include higher frequency of hypertensive disorders, (including preeclampsia), thromboembolic events, fetal demise, fetal growth restriction (FGR), preterm birth, 12 and a higher risk of maternal death. 11 13 Also, sickle cell complications, such as pain episodes, acute chest syndrome, anemia, and infections 14 15 are common in pregnant women, 12 and responsible for recurrent hospitalizations and morbidity.

## Pathophysiology of Sickle Cell Disease

The polymerization of deoxyhemoglobin S upon exposure to low oxygen levels deforms the membrane of red blood cells, which become elongated in the typical sickled shape, more adherent to endothelial cells, and less flexible. 8 The same changes can also occur in reticulocytes. 16 Red blood cell sickling shortens the lifespan of hemoglobins by removing them from circulation either by the reticuloendothelial system or intravascular hemolysis. 8 The release of free hemoglobin from within red blood cells, along with sickling and cell adhesion to endothelial cells, leads to endothelial activation, generates cytokines, and activates the coagulation cascade in a systemic inflammatory response. 17 Neutrophils and platelets are also activated, ultimately culminating in sickle cell vaso-occlusion, 8 16 a hallmark of the disease, which is responsible for acute complications as well as end organ damage due to ischemia and inflammation. 8 In the past 30 years, major advances in research have resulted in a better understanding of the pathophysiology of SCD, and earlier interventions during childhood, including the use of penicillin, vaccinations, screening and prevention of stroke, for example, have improved the quality of life of patients and have extended the life expectancy of people with SCD, enabling women to reach childbearing age 18 19 20 21 and pursue pregnancy. Sickle cell disease is a chronic inflammatory condition, and pregnancy in such women is considered a high-risk condition 14 22 23 24 25 26 that requires close follow up in specialized medical services with a multidisciplinary approach.

## Clinical and Obstetric Complications in SCD

Several studies 1 14 15 have reported maternal complications of SCD, including vaso-occlusive crises (VOCs, the most frequent cause of morbidity and hospitalization), infections (especially in the urinary tract), thromboembolic events (including deep venous thrombosis and stroke), pulmonary complications (the main cause of mortality), chronic renal failure, cesarian section, and maternal death. Fetal complications include FGR, low birth weight (LBW), prematurity, fetal distress during labor, and increased perinatal mortality. 10 14 15 22 27 However, there is a great deal of variation among published reports regarding the incidence of complications due to different study designs, country or world region of the studied cases, access to healthcare, and even the absence of statistical analysis to account for confounding variables. 12 A meta-analysis published in 2015 10 compared maternal and perinatal outcomes among women with and without SCD. The study analyzed 26,349 women with SCD and compared them in groups according to the genotype (1,276 women with HbSS and 279 women with HbSC); however, the majority of SCD cases had no known genotype (24,794). The study reported an almost 18-fold increase in maternal mortality among women with SCD compared with non-SCD women, and a more than 2-fold risk of developing preeclampsia. No additional risk of developing eclampsia was found, except among those women with the HbSS genotype (an almost 5-fold risk). The authors 10 also reported a small increase in the risk of undergoing cesarean section in all SCD groups, and a higher risk of having stillbirth in both studied genotypes. Neonatal death and premature birth were twofold higher in the HbSS group only, and small for gestational age babies were more prevalent among SCD women than among the control group. 10 A 2010 study 27 on mortality in SCD and the use of hydroxyurea (the first drug approved to treat SCD by increasing fetal hemoglobin production) showed a reduction in SCD complications such as painful episodes, blood transfusion, and acute chest syndrome, increasing the chance of patient survival from 65% to 86% in the from 65% to 86% in the treated group. 27 Although hydroxyurea is not approved for use in pregnancy, its use may be considered after pregnancy and breastfeeding in order to improve quality of life and reduce hospitalizations. Another study, 28 published in 2018, which evaluated adverse outcomes among patients with different SCD genotypes (HbSS, HbSC, and HbSβ thalassemia), included 89 women and found that 52% were hospitalized during pregnancy for clinical or obstetrical complications. The main reasons for hospitalization were VOC (41%) and obstetric adverse events (22%), and most of them occurred in HbSS patients. However, the authors 28 did not find statistically significant differences among the SCD genotypes. Perinatal outcomes such as LBW, prematurity, preeclampsia, and stillbirth were more frequent in the HbSS and HbSC groups, with no significant difference among the SCD groups. Postpartum adverse outcomes (hemorrhage, infections, and thromboembolic events) were significantly more frequent among the HbSβ thalassemia group (57%) compared with the HbSS (18%) and HbSC (13%) groups. 28 Another study, 29 with 62 SCD pregnancies, compared the complications within the 3 genotypes (HbSS, HbSC, and HbSβ thalassemia). Urinary tract infection was the most common complication, with similar frequencies in the HbSS and HbSC groups (30% and 33% respectively). The second most prevalent complication was VOC, and it was more frequent among HbSC pregnancies (27%). Preeclampsia occurred in 11% of cases of HbSS, in 40% of cases of HbSβ, and in 20% of HbSC pregnancies. Cesarian section was the delivery mode in 37%, 70%, and 40% of the cases in the HbSS, HbSC, and HbSβ thalassemia groups respectively. The rates of prematurity were of 41% in the HbSS group, and of 23% in the HbSC group, with no reported cases among the HbSβ thalassemia group. Stillbirth only occurred among the HbSS group (11%), and no maternal deaths were reported in the study. 29 In Nigeria, a study 24 compared 50 HbSS women with normal controls, and the results showed a higher frequency of pregnancy-induced hypertension (28% in the HbSS group and 6% the in control group) and 32% of VOC in the SCD group. The authors 24 also found 16% of patients with FGR and no cases in the control group. Preterm delivery was also more frequent in the HbSS group (28% versus 10%). Overall, complications were significantly higher in the HbSS group, occurring in 92% of the women (versus 38% in the controls). 24 The high frequency of complications in SCD pregnancies corroborates the need for early diagnosis and surveillance to reduce morbidity and mortality. Maternal mortality ranges from 1% in a retrospective study conducted in the United States 30 to 9.2% in a Nigerian study. 31 That discrepancy may be due to differences in the quality of care, including early diagnosis and treatment of complications, as well as under-reporting of cases. Near miss is a condition in which women survive a severe complication during pregnancy, childbirth, or within 42 days of the postpartum period. In Latin America, it is estimated there are 34 cases of near miss to every one thousand live births, 32 and 15 cases of near miss for each maternal death. 33 34 About a third of SCD pregnant women face a near miss event during pregnancy or the puerperal cycle, especially due to acute chest syndrome, a severe form of VOC affecting the lungs, and the leading cause of death in adult SCD patients. 27 35 Unsurprisingly, SCD increases the possibility that a woman will experience a near miss event during pregnancy and/or the postpartum period. Despite the acute maternal severity of a near miss condition, this event is also associated with LBW and very low birth weight newborns, newborn admission to intensive care, stillbirth, early neonatal death, and long maternal hospital stays. 32 36 37 38 Studies with SCD in pregnancy usually involve a small number of patients due to the relative rarity of the condition and the difficulties in the compilation of data. A summary of meta-analyses on SCD during pregnancy found in the PubMed, SciELO, and Embase databases is presented in Table 1 .

**Table 1:** Systematic Reviews on Sickle Cell Disease during Pregnancy

**Author (year, country)**	**Study design**	**Number of studies and/or women**	**Outcomes**	**Conclusions**
Oteng-Ntim et al. 10 (2015, United Kingdom)	Meta-analysis	26,349 SCD women	MM (RR: 18.51; 95%CI: 8.63–39.72) PE (RR: 2.06; 95%CI: 1.49–2.85) CS (RR: 1.27; 95%CI: 1.18–1.36) ND (RR: 2.68; 95%CI: 1.49–4.82)	Pregnant women with SCD have high risks of developing maternal and perinatal adverse outcomes; risks are greatest for those in low-income countries and for those with HbSS disease compared to HbSC
Boafor et al. 37 (2016, Ghana, Nigeria, and United States)	Meta-analysis	- 9 studies	MM (OR: 10.91; 95%CI: 1.83–65.11; *p* = 0.009)	SCD increases the risk of adverse maternal and perinatal outcomes in low- and high-income countries
		- 12 studies	PE (OR: 2.05; 95%CI: 1.47–2.85; *p* < 0.001)	
		- 13 studies	CS (OR: 1.54; 95%CI: 1.27–1.87; *p* < 0.001)	
		- 6 studies	ND (OR: 2.71; 95%CI: 1.41–5.22; *p* < 0.003)	
		- 10 studies	FGR (OR: 2.69; 95%CI: 1.85–4.21; *p* < 0.001)	
		- 6 studies	Perinatal mortality (OR: 3.76; 95%CI: 2.34–6.06; *p* < 0.001)	
		- 11 studies	Prematurity (OR: 2.14; 95%CI: 1.56–2.95; *p* < 0.001)	
		- 9 studies	LBW (OR: 2.00; 95%CI: 1.42–2.83; *p* < 0.001)	
		- 10 studies	Stillbirth (OR: 4.05; 95%CI: 2.59–6.32; *p* < 0.001)	
		- 6 studies	Infection (OR: 2.48; 95%CI: 1.23–5.01; *p* = 0.011)	
Inparaj et al. 38 (2020, United Kingdom)	Meta-analysis	3,964 Patients	ACS/pneumonia (event rate: 6.46%; 95%CI: 4.66–8.25); Pulmonary thromboembolism (RR: 7.74; 95%CI: 4.65–12.89)	Strong association between SCD and maternal pulmonary complications

Abbreviations: 95%CI, 95% confidence interval; ACS, acute chest syndrome; CS, cesarian section; FGR, fetal growth restriction; LBW, low birth weight; MM, maternal mortality; ND, neonatal Death; OR, odds ratio; PE, preeclampsia; RR, risk ratio; SCD, sickle cell disease.

### Antenatal Care

Since SCD is a chronic systemic condition, pregnancy should ideally be planned in order to minimize possible complications. One main concern is regarding the use of medications for SCD during pregnancy and lactation. Common medications used in SCD management that should be discontinued before and during pregnancy include angiotensin-converting enzyme (ACE) inhibitors, iron chelators, and hydroxyurea. The use of hydroxyurea is not recommended, and women are advised to avoid conception up to 6 months after their last dose of hydroxyurea due to animal studies showing teratogenicity, and a few case reports of fetal growth abnormality and preterm birth. For more recently-approved SCD-specific therapies, the antioxidant aminoacid L-glutamine is generally considered to be safe, the antisickling agent voxelotor is safe through pregnancy but not recommended during lactation, and there are no data on the anti-adhesive monoclonal antibody crizanlizumab. Care for pregnancy in SCD patients must include specialized antenatal care, since complications can occur at early gestational ages. Pregnancy in SCD patients requires more frequent follow-up, including a multidisciplinary team 22 26 39 with an obstetrician, with close fetal surveillance, hematological support, nutritional and psychological assessments, and an ultrasonographist experienced in materno-fetal medicine. It may be convenient that appointments with the obstetrician take place at least monthly in the first and second trimesters, and more frequently after that, with individual assessment. It is paramount to check the immunization status and update it when necessary, taking into consideration that SCD patients require broader coverage for encapsulated bacteria (such as *Streptococcus* *pneumoniae* , *Haemophilus influenzae* , and *Neisseria* ). Laboratory checks must include hemoglobin level, hemolytic markers (reticulocyte count and lactate dehydrogenase [LDH]), and screening for infections (especially urinary tract infection and asymptomatic bacteriuria), more frequently than for a low-risk pregnancy. Anemia during pregnancy is defined by the World Health Organization (WHO) as a hemoglobin level below 11g/dL 40 41 at any trimester of gestation. Maternal anemia is a common condition even for women with normal hemoglobin, especially during the last trimester of pregnancy, when the levels of iron need to be increased. 42 When anemia presents in the first and second trimesters, it may contribute to LBW, prematurity, and neonatal complications. 42 43 44 Chronic anemia is a very common feature of SCD, and it impacts patients since the beginning of pregnancy. However, it is important to assess if there are additional contributors to maternal anemia, such as nutritional deficiencies. Anemia can negatively affect fetal development, resulting in a growth-restricted fetus. 43 44 45 46 The hemoglobin level of these patients can be very low, and the need for blood transfusion is common. Therefore, many studies have evaluated the benefits of prophylactic blood transfusion during pregnancy. Transfusion of red blood cells aims to improve the oxygen-carrying capacity. It can be performed through simple transfusion, when the goal is to achieve a certain level of hemoglobin, or exchange transfusion (manual or automated erythrocytapheresis), 47 when the aim is to decrease the levels of circulating HbS. In acute complications that lead to severe anemia, simple transfusion is usually the choice. Those transfusions aim to increase hemoglobin, taking it to levels closer to baseline while avoiding hyperviscosity and heart failure. 8 Red blood cell transfusions can also be chronically used for stroke prevention, and acutely to treat acute chest syndrome or multiorgan failure. 8 Exchange transfusion has been used during pregnancy to reduce the occurrence of complications, but there is no definitive evidence to indicate it to all patients. If red blood cell exchange is indicated in pregnancy, reasonable targets would be a level of HbS lower than 40%, with a level of hemoglobin of 10g/dL. 48 A meta-analysis published in 2015 49 concluded that prophylactic transfusion was associated with lower maternal mortality, VOCs, pulmonary complications, perinatal and neonatal mortality, and premature delivery, but no improvement in preeclampsia and FGR. Other studies reinforce the findings of lower maternal mortality 50 and VOCs 50 51 with prophylactic transfusion. A 2007 Brazilian study 52 found better fetal outcomes with fewer cases of FGR and preterm deliveries. Nevertheless, there is no consensus regarding the ideal hemoglobin level during pregnancy or the best moment to start transfusions in pregnant SCD patients. Some authors 50 recommend starting transfusions in the second trimester while others 53 54 recommend an earlier start, in the first trimester. A more conservative approach is to transfuse pregnant patients who have severe anemia with Hb below 7g/dL, or any level of anemia if signs of impaired fetal growth or fetal distress are observed. To make this decision, providers should also consider that the more transfusions, the greater the risk of alloimmunization, which is of about 16% to 20% in this population. 26 50 Alloimmunization can lead to lifetime difficulty to find compatible blood and delayed hemolytic transfusion reactions, 55 the latter sometimes manifesting with hyperhemolisis syndrome, a life-threatening situation in which hemoglobin levels drop to below pretransfusion levels.

## Placental Findings in SCD

The placenta is an organ with an adapted surface for oxygen and nutrient exchange between the maternal and fetal circulations. 56 Studies on SCD 57 58 have shown placental abnormalities such as syncytial knots, villous necrosis, congestion, deposits of sickle hemoglobin, and intravillous fibrin. Increased expression of proinflammatory genes in the placenta have been documented, suggesting that the organ is exposed to a proinflammatory environment and hypoxia, 46 and the imbalance in inflammatory substances could favor vaso-occlusive episodes and necrosis. 48 57 Furthermore, abnormal placental size, location, and adhesion to the uterine wall have also been described in SCD pregnancies. The exact pathophysiology explaining how abnormalities in placental development can contribute to worse perinatal outcomes and complications in SCD is not yet fully understood. However, those abnormalities may increase the risk of uteroplacental insufficiency, leading to maternal and fetal adverse outcomes. 46

## Fetal Surveillance and Risk of Fetal Growth Restriction

Fetal growth restriction occurs when the fetus does not reach its biological growth potential and is usually associated to placental insufficiency. 59 Those fetuses are at a higher risk of adverse outcomes in pregnancy, higher morbidity and mortality, and impaired neurological development, 60 61 which makes recognition and appropriate follow-up of such cases essential. Statistical deviations from population-based reference growth curves define FGR. 62 For fetal assessment, the ultrasound (US) scan is the preferred method to evaluate fetal wellbeing, as it can estimate fetal weight (which is especially important during the third trimester) and detect placental impairment with Doppler velocimetry; therefore, it should be part of the regular antenatal care. However, due to the heterogeneity among SCD patients, there is no specific recommended protocol for US follow up. We suggest performing US at least once during the first and the second trimesters, and then monthly until delivery. That seems like a reasonable approach if there is no major complication, and providers should consider shorter scan intervals if necessary. During the third trimester, closer follow-up with a two-week interval if there is early suspicion of fetal impairment is acceptable.

## Diagnosis and Treatment of Complications during Pregnancy

The complications of SCD may have distinct presentations and should always be suspected based on patient's history and examination. The symptoms may mimic common pregnancy discomforts, delaying adequate healthcare. I most frequent complication is VOC, which is typically experienced as acute bone or joint pain that starts abruptly. Uncomplicated VOCs last for four to five days on average. The severity of the pain also varies widely, but severe pain often requires admission. The treatment of VOC is based on hydration, analgesia, and treatment of the precipitating factors. Common triggers for VOC include dehydration, sudden changes in temperature (including but not limited to cold exposure), infections (including urinary tract infection, pneumonia, acute osteomyelitis etc.), delayed transfusion reactions, thromboembolic events, the acute phase of avascular necrosis, and emotional distress, but many VOCs will not have an identifiable trigger. Controlled fluid management should be enough to reduce blood viscosity, but must not be overdone to avoid acute pulmonary edema. There is no scientific evidence to recommend a specific type of intravenous fluid, and providers can freely choose to use normal saline, sodium chloride (NaCl) 0.45%, Ringer lactate slution, and others. Analgesia must include non-opioid and/or opioid analgesics, depending on the intensity of the pain. 63 The use of opioids during pregnancy increases the risk of neonatal complications, including withdrawal syndrome in the newborn, but should not be considered a contraindication to the use of opioids in this setting. Non-steroidal anti-inflammatory drugs are not recommended after 34 weeks gestation in order to avoid cardiac dysfunction with premature closure of the arterial canal. Transfusions should not be routinely indicated for VOCs, and should take into consideration if there is symptomatic anemia, and the potential risks associated with the procedure. Acute chest syndrome is a major complication characterized by a VOC with acute pain in the thoracic region associated with respiratory symptoms and fever, mostly associated with the finding of a new pulmonary opacity, and hypoxemia in severe cases. It is the main cause of death among adults with SCD. 10 The treatment is symptomatic, with analgesia, fluid management, oxygen supplementation for oxygen saturation below 92%, and ventilatory support if necessary. Simple transfusion may be indicated in patients with severe anemia (Hb < 7g/dL), and red blood cell exchange transfusion must always be considered for severe cases with hypoxemia. Acute chest syndrome is indistinguishable from pneumonia; therefore, the use of empiric broad-spectrum antibiotics is indicated, antivirals should be considered, and consultation with an infectious disease's specialist is encouraged to discuss options during pregnancy. The association of SCD and pregnancy increases the risk of deep-vein thrombosis and pulmonary embolism, so a low threshold to indicate lung computed tomography (CT) angiography is encouraged for patients with signs and symptoms of acute chest syndrome. While preeclampsia is known to be more frequent in SCD patients, the diagnosis of severe preeclampsia with hemolysis, elevated liver enzymes, and low platelet count (HELLP) syndrome may be challenging. Laboratory abnormalities caused by SCD delay the detection of hemolysis, since the levels of LDH and aspartate aminotransferase (AST) are increased by chronic hemolysis, and haptoglobin is already undetectable in SCD. In addition, HbSS patients may have a baseline elevated platelet count due to autosplenectomy, sometimes above 600 thousand, and HbSC or HbSβ patients often have splenomegaly, leading to mild to moderate chronic thrombocytopenia in the 80 thousand to the 150 thousand range. Young patients may already have some degree of microalbuminuria prior to pregnancy, so early evaluation of the urinary sediment is encouraged as a baseline to help detect abnormal proteinuria later in pregnancy. Therefore, patients presenting with signs and symptoms suggestive of severe preeclampsia, such as headache and abdominal pain, should be carefully evaluated and monitored, and their laboratory evaluation must take into consideration their earlier results. We recommend keeping track of baseline urinalysis, LDH, liver enzymes, and platelet counts and throughout pregnancy to help the diagnosis of preeclampsia and HELLP syndrome in SCD patients. Sickle cell disease complications such as sickle hepatopathy and hepatic sequestration should be considered differential diagnoses, since they may also lead to an increase in the levels of liver enzymes and worsen the anemia.

## Follow-up and Childbirth

**Fig. 1.**
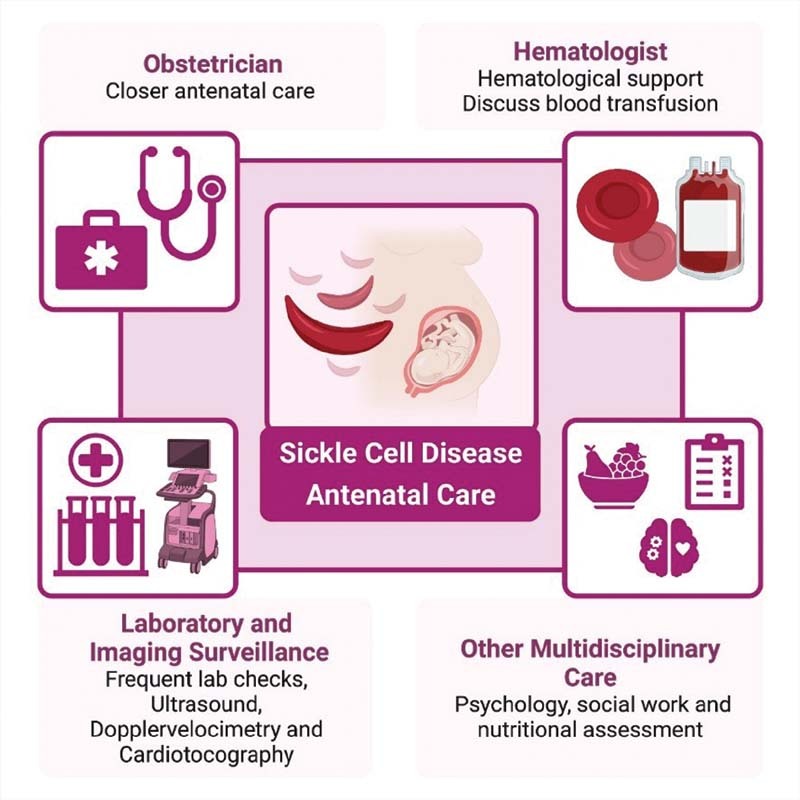
Antenatal care of pregnant women with sickle cell disease (SCD).

**Fig. 2.**
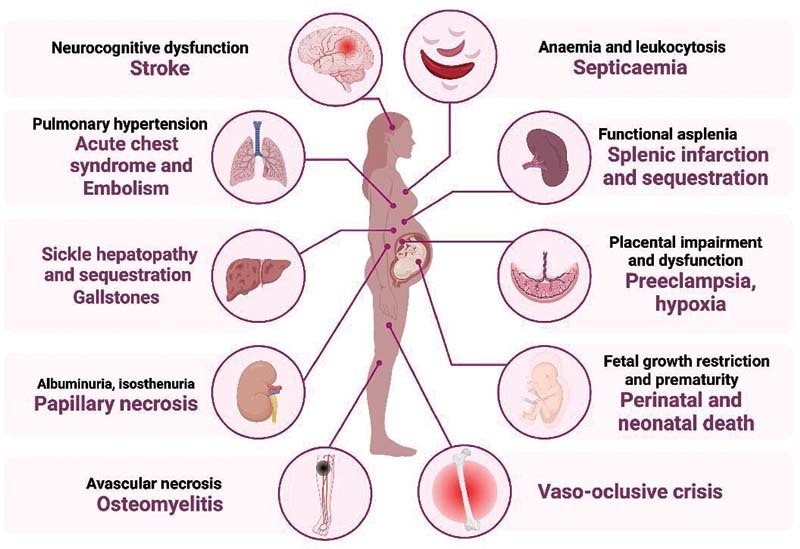
Frequent complications in SCD.

Vaginal delivery is possible, so SCD should not be regarded as an indication for cesarian section. Labor may occur spontaneously or after induction in SCD pregnant patients. During labor, it is important to be mindful of the patient's hydration status, provide appropriate analgesia, since those women are more prone to vaso-occlusive events during stressful situations, and ensure close fetal monitoring. Since SCD is a chronic systemic condition, pregnancy in SCD patients should ideally be planned in order to minimize possible complications. Women of reproductive age and those in the postpartum period must be counseled about contraceptive options. According to the WHO, contraceptive methods containing only progesterone (the pill, injectable contraceptives, implants, or intrauterine devices) are category 1, meaning that the method can be used in any circumstance. Combined hormonal contraceptives and copper intrauterine devices are category 2, meaning that the method can generally be considered, but should take into account the individual risk of thromboembolic events, the history of menorrhagia, and patient preference. 64 Psychological and nutricional support should happen together with antenatal and postpartum care, as these patients have higher rates of hospitalization and are more prone to develop depressive episodes 65 66 and chronic nutritional deficiency. 67 Sickle cell disease does not constitute a contraindication for breastfeeding, but the treatment with hydroxyurea is usually postponed until the patient is not breastfeeding. In addition, patients who were treated with blood transfusions during pregnancy should be evaluated for iron overload by their hematologist, particularly if chronically transfused. The indication for iron chelation therapy will depend on workup with serum ferritin, transferrin saturation, and liver magnetic resonance imaging. Low bone mineral density is a frequent complication of SCD, 68 even in women of childbearing age, and may remain undiagnosed for many years. Pregnancy and breastfeeding contribute to deplete calcium from the bones, 69 and we encourage providers to evaluate patients for osteopenia and osteoporosis with a dual-energy X-ray absorptiometry (DEXA) scan after they have finished breastfeeding, and verify vitamin D levels to provide supplementation if needed ( Figures 1 and 2 ).

## Conclusion

Sickle cell disease is a complex chronic disorder with potential life-threatening complications during pregnancy. The management of pregnant SCD patients requires a multidisciplinary approach to achieve favorable maternal and fetal outcomes, with accurate and timely diagnosis and treatment of its complications.
